# Obtaining Ultrafine Dispersions of Silver Particles in Poly(vinyl Alcohol) Matrix Using Mechanical Alloying

**DOI:** 10.3390/polym14173588

**Published:** 2022-08-30

**Authors:** Deize Basílio dos Santos de Aguiar, Denilson José Marcolino de Aguiar, Josiane de Fátima Padilha de Paula, Osvaldo Mitsuyuki Cintho

**Affiliations:** 1 Departamento de Estética e Cosmética, Faculdade Cesumar de Ponta Grossa (Unicesumar), Ponta Grossa 84036–350, PR, Brazil; 2 Departamento Acadêmico de Mecânica (DAMEC), Universidade Tecnológica Federal do Paraná—UTFPR, Ponta Grossa 84017–220, PR, Brazil; 3 Department of Pharmaceutical Sciences, State University of Ponta Grossa, Ponta Grossa 84030–900, PR, Brazil; 4 Departamento de Engenharia de Materiais (DEMA), State University of Ponta Grossa, Ponta Grossa 84030–900, PR, Brazil

**Keywords:** silver nitrate, silver particles, poly(vinyl alcohol), mechanical alloying

## Abstract

Mechanical alloying was performed to obtain a composite material with a homogeneous dispersion of silver particles in a poly(vinyl alcohol) (PVA) matrix. Silver is a bactericidal material, and PVA is a widely used biocompatible polymer. Therefore, this mix can lead to a potentially functional biomaterial. This study focuses on the combination of both materials, processed by mechanical alloying, which has a promising application potential. The silver (Ag) used was ultrafine, measuring between 200 and 400 nanometers, produced from silver nitrate (AgNO_3_) redox. The Attritor high–energy, water–cooled ball mill was used to mill PVA for 4 h, at 600 rpm speed rotation and 38:1 power milling. Mechanical alloying was demonstrated to cause particle refinement in PVA with a timespan of 1 h. A slight additional particle decrease occurred for long–time milling. A milling time of 4 h was used to disperse the silver particles in the polymer matrix homogeneously. Hot pressing films were produced from the obtained dispersion powders. The microstructural features were studied using several material characterization techniques. Antimicrobial Susceptibility Tests (AST), conducted in an in–vitro assay, showed a hydrophilic character of the films and a protection against bacterial growth, making the process a promising path for the production of surface protective polymeric films.

## 1. Introduction

Nowadays, mechanical alloying is studied in all materials. It ranges from metals and their alloys to obtaining several composite materials involving metals, polymers, and ceramics to produce various commercial application materials of scientific interest. Among the primary purposes of this technique is to obtain: the formation of alloys in solid state; the possibility of obtaining alloys with immiscible elements by other methods; the refinement of grain size to nanometric levels; the decrease or increase in particle size; particle agglomeration; extension of solubility limits; the development of amorphous phases; and the induction of chemical reactions at low temperature, among others [1,2,3]. Mechanical alloying is a solid–state process that consists of adding the materials to be ground, usually dry, together with the grinding balls inside a jar. The intense impact between the material, the spheres, and the jar walls produce a fine powder with a much higher energy than that of other standard grinding processes [1,2]. The application of this technique in polymeric materials is more recent than in other materials, beginning in the late 1980s [4,5]. However, in the last decade, the production of polymer matrix composites in several applications has become widely studied [3,6,7,8,9,10]. The mechanisms acting in the polymer mechanical alloying are somewhat different from other materials. There is the possibility of chain breakage during collisions between the samples and the grinding media, leading to a decrease in molar mass. Nevertheless, breaking bonds in the polymeric chains can also lead to free radicals due to fracture processes, providing new chemical bonds and, thus, mixing immiscible polymers in other ways, forming blends without the solvents used [4,5,11,12,13,14,15]. Furthermore, the crystal structure of semi–crystalline polymers can be altered by milling [6,12]. 

Commonly, polymer matrix nanocomposites require several processing steps, and the processes are slow. Some of them take days to manufacture [16,17,18,19,20]. The product’s final volume is usually small, limited by the nature of the process. The sol–gel [21] and the solution–based, self–assembly (layer–by–layer) methods are highlighted [22,23,24,25,26]. The sol–gel method synthesizes materials from the sol system transition to a gel system. The term sol defines a colloidal particle dispersion (sizes between 1 and 100 nm). In contrast, the term gel consists of a system formed by the rigid structure of colloidal particles (colloidal gel) or polymeric chains (polymeric gel), immobilizing the liquid phase inside the interstices [21,27]. The solution is the self–assembly (layer–by–layer) technique in a liquid medium, which results in self–structured layers. It consists of the deposition of several layers adhered by the electrostatic attraction between the opposite charges of their constituents. The layers’ deposition is based on physical adsorption, innovating another existing technique based on chemical adsorption [23,24,25,26]. 

Comparatively, high–energy ball milling is a solid–state method that does not require additional solvents and all production steps can be made in a dry state. In addition, the only limitation to the bulk material’s production quantity is the jar’s volume. However, there are industrial jars capable of grinding large volumes of material in one run, making mechanical alloying an appealing and possibly viable technological path to obtain polymer matrix composites [1,2]. 

PVA is a synthetic polymer that is thermoplastic, semi–crystalline, non–toxic, biocompatible, biodegradable, hydrophilic, and belons to the polyvinyl ester class. It is commercially available at a low cost, being one of the few water–soluble polymers, depending on the temperature. Its melting temperature is between 180 and 230 °C, depending on the crystallinity degree [28,29,30,31,32,33]. PVA has an excellent ability to form films and hydrogels. In addition, the hydrophilic characteristic of PVA can protect a region from moisture, as it exhibits a high level of absorption of water or biological liquids. Because of this characteristic, PVA can form films with several functionalities, including simulating a natural tissue, enabling numerous applications in biological materials [28,32,33,34]. Silver (Ag) has been used due to its potent antimicrobial activity properties against various types of pathogens, especially in recent times due to the emergence of resistant bacteria [35,36,37,38,39,40,41,42,43,44,45]. Furthermore, when it is combined with PVA, a biocompatible and biodegradable polymer, it is possible to obtain a polymeric film, resistant to microbial contamination, ideal to create films for potential application as biomaterials [34,46,47,48,49]. The originality of this work lies in the use of mechanical alloying in the production of the PVA/Ag composite, which has proved to be a promising processing path. This powder can be easily formed to produce bacterial growth–inhibiting films. Such films can be used both in biocompatible functions and in other applications that require the absence of microorganisms. 

The present work is aimed at studying the feasibility of using mechanical alloying to obtain a composite material with an ultrafine dispersion of Ag particles in a PVA matrix using an Attritor mill, with subsequent evaluation of the antimicrobial activity of the obtained materials.

## 2. Materials and Methods

The materials used in this research were:

- Silver nitrate (AgNO_3_)—in powder form, white, supplied by the company Sigma–Aldrich, Saint Louis, MO, USA. It has a silver content of 63.35% and 99.98% purity.
- Poly(vinyl alcohol) (PVA)—in the form of a white, odorless granular powder, supplied by the Chemical Company, Jamestown, RI, USA, with an approximate molar mass of 72 × 103 g mol^−1^ and a hydrolysis degree of 88.09%.
- Sodium hydroxide (NaOH) in powder form, white, supplied by the company Sigma–Aldrich, Saint Louis, MO, USA with an approximate molar mass of 40 g mol^−1^.
- Formaldehyde (HCOH) supplied by Dinâmica Química Contemporânea LTDA, Indaiatuba, SP, BRA, with an approximate molar mass of 30.03 g mol^−1^.

In this research, obtaining Ag was possible by using consecutive oxidation reactions and reduction of silver nitrate [50,51]. Mechanical alloying was used to homogeneously disperse the Ag particles in the polymeric matrix. With the obtained dispersion, films were produced by the widely used hot pressing method [52,53]. The samples were characterized by X-ray diffraction, optical microscopy, scanning electron microscopy by field emission electron gun *(SEM–FEG)*, and differential and infrared exploratory calorimetry (*DSC* and *FTIR*) analysis.

### 2.1. Chemical Reactions to Obtain Silver

Firstly, a 250 mL of sodium hydroxide solution (NaOH) with a concentration of 1 molar was prepared in a volumetric flask. Afterwards, 10 g of white silver nitrate (AgNO_3_) was weighed in a beaker. The NaOH solution was subsequently added to the beaker with the AgNO_3_ and kept at 80 °C on a Solab magnetic stirrer with a heating plate (model SL–91) to homogenize the solution and accelerate the reaction for forming black–colored silver oxide (Ag_2_O), according to Equation (1). After 40 min under the conditions described, the heating plate was switched off. The resulting residue was placed on filter paper, washed in distilled water several times to remove residues from the solution, and dried to perform X-ray diffraction and *SEM–FEG* analysis of Ag_2_O. After that, the Ag_2_O formation experiment was repeated, and about 10 mL of formaldehyde (HCOH) was slowly pipetted for the formation of yellowish silver (Ag) to occur (as it is unpolished), with some metallic shine points according to Equation (2). The resulting residue was also placed on filter paper, successively, and several rounds of rinsing was conducted followed by oven drying to perform an X-ray diffraction and an *SEM–FEG* analysis of Ag.

The expected reactions were as follows:

(1) $$2Ag^{+}+2NO_{3}{}^{-}+2Na^{+}+2OH^{-}\to \;Ag_{2}O↓+2NaNO_{3}+H_{2}O$$

(2) $$Ag_{2}O+HCOH+H_{2}O\to 2\;Ag↓+CO_{2}+4\;H_{2}O$$

The filtering was performed in a device connected to a vacuum pump and later placed in an oven at 40 °C for 24 h for drying purposes. Afterwards, they were photographed, and their visual aspects compared with the as–received sample.

### 2.2. X-ray Diffraction of Silver Nitrate, Silver Oxide, and Silver

The characterization was performed in a Rigaku Diffractometer, model Ultima IV, using Cu–Kα radiation (0.154 nm). The equipment is installed in the Multi–User Laboratory (C—LABMU) of the State University of Ponta Grossa. The scanning ranges (diffraction angle 2θ) were from 20 to 90° in the step scan mode, with a step of 0.02° and 5 s at each point.

### 2.3. SEM–FEG of Silver Nitrate, Silver Oxide, Silver, and PVA

The samples were metalized with a gold and palladium alloy to avoid electrical charging before being placed into the microscope. The metalizing equipment used is from Qorum Sputter Coater, model SC7620. An *SEM–FEG* characterization was performed using Tescan equipment, model Mira. Both equipament are installed in the Multi–User Laboratory (C—LABMU) of the State University of Ponta Grossa. To obtain pure PVA, AgNO_3_, or composite PVA/Ag images, the *SEM–FEG* was operated between 3 and 5 kV, although 15 kV for Ag and Ag_2_O, using secondary electrons in all cases. The low voltage for AgNO_3_ and the polymer matrix was chosen because higher voltages cause charging and damage to these types of materials, respectively.

### 2.4. Commercial PVA Mechanical Alloying and SEM–FEG of Samples

The commercial PVA samples were processed with high–energy ball milling in a Union Process Attritor mill, model Attritor 01 HD, installed at the Department of Materials Engineering at the State University of Ponta Grossa. The milling balls used were made of zirconia with a 6.35 mm diameter size. The use of this type of ball instead of steel balls was to minimize contamination with traces of metal. For this same reason, the jar and milling rod used were made of ultra–high molar mass polyethylene, while the rod arms were made of zirconia. Running water at room temperature cooled the mill; thus the heat generated by the grinding action would be dissipated, avoiding possible thermal degradation of the polymer. The milling power adopted in this work was 38:1 for all milling performed. The speed rotation was 600 rpm. The milling parameters are summarized in Table 1 while the PVA milling times are described in Table 2:

After that, *SEM–FEG* sampling was performed with the same parameters and procedures indicated in the previous item, to verify the influence of milling on the size and morphology of the polymer particles.

### 2.5. Differential Scanning Calorimetry (DSC) Analysis Processed in an Attritor–Type Mill

The *DSC* analyses were performed using Setaram equipment, Labsys model, installed in the Multi–User Laboratory (C—LABMU) of the State University of Ponta Grossa, with a thermal analysis capacity of up to 1600 °C. The analysis was conducted in an argon atmosphere with a 20 mL/minute flow in an alumina crucible with approximately 30 mg of material. The heating and cooling rates were 10 °C/minute. The scanning range was from 20 °C to 230 °C, performed in a single cycle. This characterization was intended to verify the influence of different milling times on the melting temperature of the PVA, comparing it with the as–received material, as shown in Table 2.

### 2.6. Mixing of Several Amounts of PVA and Ag by Mechanical Alloying and Infrared Spectrophotometry Assay with Fourier Transform (FTIR) Analyses of the Mixtures

Some Ag dispersions were prepared in a PVA matrix, where the Ag was obtained by oxidation reactions and reduction of AgNO_3_. These mixtures were conducted in the same high–energy ball mill used to mill commercially pure PVA. Preliminary experiments were performed, such as milling together the Ag and the PVA from the beginning for 2 and 4 h. Another attempt was made to mill the PVA alone for 1 h, stopping the milling and adding the Ag and milling for another 1 h. Under these conditions, there were still many non–adhered and unsatisfactorily dispersed Ag particles on the polymer matrix. Therefore, all of these parameters were discarded. Based on the preliminary results, *DSC* and *SEM–FEG* analyses, the milling time to mixing PVA and Ag chosen was 4 h, while the milling parameters were the same as from Table 1. 

The procedure to obtain the Ag dispersion in the matrix was to mill the commercially pure PVA for 2 h, followed by an interruption of the milling and mixing of the Ag with the previously ground PVA, and the finalization of the Ag milling process for another 2 h. In this way, the best relation between the PVA milling time and Ag dispersion in the matrix of the mill operation was achieved.

Milling products with different mass concentrations of Ag weighed on an analytical balance with a mass/mass ratio were elaborated for further characterization and verification of the metal effects in the matrix. The mass fractions used in this work compared with the as–received PVA are shown in Table 3.

After that, the *FTIR* analysis was conducted using SHIMADZU equipment, model IR Prestige–21, and 64 scans/min, resolution of 4 cm^−1^, installed in the Multi–User Laboratory (C—LABMU) of the State University of Ponta Grossa. The samples analyzed were as–received PVA (A), pure PVA milled for 4 h (E) from Table 2, and powdered mixture PVA with Ag particles, from Table 3 (E1 to E5). 

The powder samples were prepared via cold pressing using a SHIMADZU manual hydraulic press, installed in the Multi–User Laboratory (C—LABMU) of the State University of Ponta Grossa. These disks were set as the sample mixture to be analyzed with potassium bromide (KBr) from the Vetec, each 5 and 195 mg. A reference reading was performed with a pure KBr disk.

### 2.7. Preparation of Polymeric Films from the PVA/Ag Mixture by Hot Pressing and Characterization by Transmitted Light Optical Microscopy

In this step, for each sample condition, films were made via hot pressing in a Schwing Siwa manual hydraulic press, model 30 tons, installed at the Department of Materials Engineering at the State University of Ponta Grossa. After several tests, the film with a more homogeneous aspect was obtained by processing at a temperature of 220 °C. Then, the press was heated to 220 °C with a thermocouple control. The films were produced with 2 g of material, resulting in a close to circular shape, 3 mm thickness, and 70 mm in diameter. The samples used to prepare films were as–received PVA (A), pure PVA milled for 4 h (E) from Table 2, and powdered mixture PVA with Ag particles, from Table 3 (E1 to E5). 

After obtaining the films, they were characterized by transmitted light microscopy to assess the distribution of Ag particles on the PVA surface. The equipment used was an Olympus optical microscope, model BX–51, installed at the Department of Materials Engineering of State University of Ponta Grossa, with a digital camera coupled and controlled by software Image–Pro Plus, version 5.1, from Media Cybernetics Inc., Rockville, MD, USA.

### 2.8. Characterization of the Fracture Surface of the Films by SEM–FEG

The films produced underwent a process of embrittlement in liquid nitrogen inside a Styrofoam container. This bath was maintained for approximately 5 min to weaken the sample and facilitate fracture, performed with standard pliers. The pieces were then metalized to produce a conductive barrier. Thus, the fracture surfaces along the thickness can be analyzed by *SEM–FEG*, mainly the distribution of Ag particles.

### 2.9. Chemical Microanalysis of Particles by Energy Dispersion (EDS)

A chemical microanalysis was conducted using an *EDS* coupled to the *SEM–FEG* equipment, device installed in the Multi–User Laboratory (C—LABMU) of the State University of Ponta Grossa. The equipment used was mentioned in the topic above. This analysis was performed to estimate the presence of Ag in the thickness of the fragile film.

### 2.10. Microbiological Sensitivity Tests on Polymeric Films

The antibiogram, also known as the Antimicrobial Susceptibility Test (*AST*), is an in–vitro assay that measures the susceptibility or resistance of bacteria to antimicrobials. The method used in this work was susceptibility in a culture plate (90 mm in diameter and 15 mm high) on Müeller Hinton agar. This medium contains proteins and carbohydrates that provide the ideal substrate for developing and growing bacterial strains of interest. This test was conducted to evaluate the antimicrobial activity of polymeric films [42,43,45,46,49,54] and it was performed at Department of Pharmacy and Biochemistry at the State University of Ponta Grossa. The analyzed samples are shown in Table 4.

The tests were conducted using bacterial strains provided by the American Type Culture Collection (*ATCC*). They are:

✓ *Escherichia coli—ATCC 0022*;
✓ *Staphylococcus aureus—ATCC 0023*;
✓ *Streptococcus pyogenes—ATCC 0015*;
✓ *Pseudomonas aeruginosa—ATCC 0016*.

The bacterial suspensions were prepared by adding some microorganism colonies in a sterile saline solution. Afterwards, the suspension was read until a turbidity of 1.5, McFarland scale (approximately 108 CFU/mL), was achieved on a Biosystems spectrophotometer, model BTS–330. This procedure was performed individually for each studied bacterium [32,35,37,41,42,44,45,46,47,48,49,50,54,55].

At that moment, each bacterial suspension was sown with the aid of a sterile swab in a culture dish. Then, 6 mm diameter polymeric film discs were deposited and dispersed on the seeded plates. These plates were incubated in a QUIMIS microbiological oven, model Q316M4, at 36 °C for 24 h. Subsequently, the plates were observed to identify the presence or absence of a bacterial inhibition halo in the culture medium. The bacteria growth inhibition halo *(H)* was measured according to Equation (3) [46], as follows:

(3) $$H=\frac{D-d}{2}$$

where *D* is the diameter of the zone of inhibition and *d* is the diameter of the PVA film disc containing Ag particles.

## 3. Results and Discussion

### 3.1. Chemical Reactions of Materials Containing Silver

**Figure 1:**
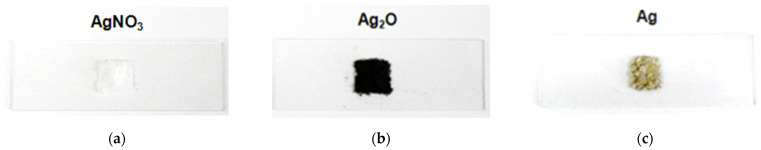
Images of as–received AgNO_3_ (**a**); precipitated from Equation (1) Ag_2_O (**b**); and precipitated from Equation (2) Ag (**c**).

Changes occurred in the coloring in the transition from as–received material to the oxidation and reduction reactions products. Initially, the as–received ANO_3_ is white. After the first reaction described in Equation (1), it adopts a black color due to the oxidation of the material, where Ag_2_O was produced. Finally, after the second reaction described in Equation (2), there was a reduction in the oxide, adopting a yellowish color. Figure 1 illustrates the color change from AgNO_3,_ passing by Ag_2_O oxidation and then reduction.

Figure 1a–c shows a color change after the reaction steps from the as–received material. Initially, as–received AgNO_3_ is white (Figure 1a), and after the first reaction described in Equation (1), it adopts a black color due to its oxidation, evidencing Ag_2_O formation (Figure 1b). Finally, after the second reaction described in Equation (2), the oxide was reduced, adopting a yellowish color due to Ag formation (Figure 1c).

### 3.2. X-ray Diffraction of Silver Nitrate, Silver Oxide, and Silver

**Figure 2:**
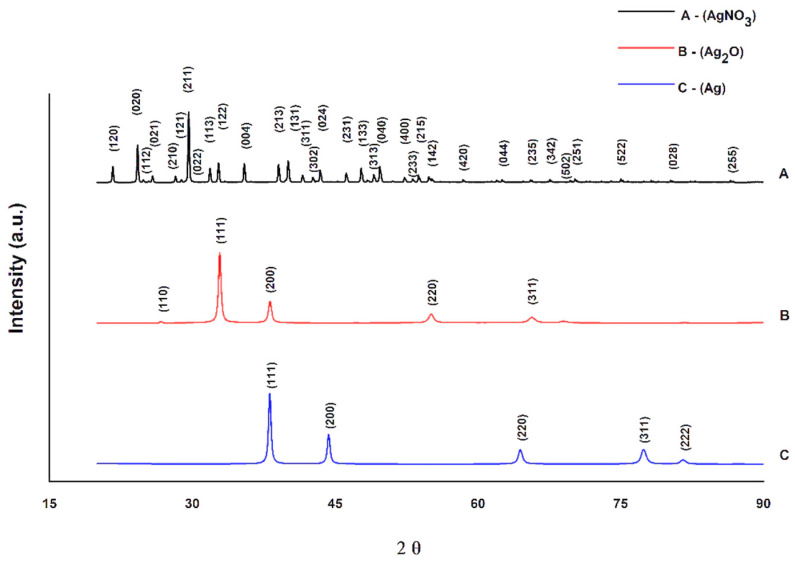
Diffractogram from silver nitrate (AgNO_3_) as–received samples, silver oxide (Ag_2_O), and silver (Ag).

The solids were characterized by X-ray diffraction, with as–received silver nitrate (AgNO_3_), silver oxide precipitate (Ag_2_O), and final solid samples silver (Ag). These changes in crystalline structures confirm the peaks of the crystallographic planes of the studied specimens, illustrated in Figure 2.

The summarized values of lattice parameters of the structures are shown in Table 5.

The results of the diffractogram analyses in Figure 2 demonstrate that the proposed reactions occurred. As–received silver nitrate has a crystalline structure with the Miller indices belonging to the orthorhombic system of the Pbca type spatial group. After the first reaction (Equation (1)), there was a change in the crystalline structure, resulting in a material with a crystalline structure with the Miller indices belonging to the simple cubic system, of the spatial group of the type Pn–3m, in the case of silver oxide. The second reaction (Equation (2)) promotes a new change, resulting in a material with a crystalline structure with Miller indices belonging to the face–centered cubic system, of the Fm–3m type spatial group, in the case of Ag. The lattice parameters presented in the Table 4 show these changes.

### 3.3. SEM–FEG of Silver Nitrate, Silver Oxide, Silver and PVA

**Figure 3:**
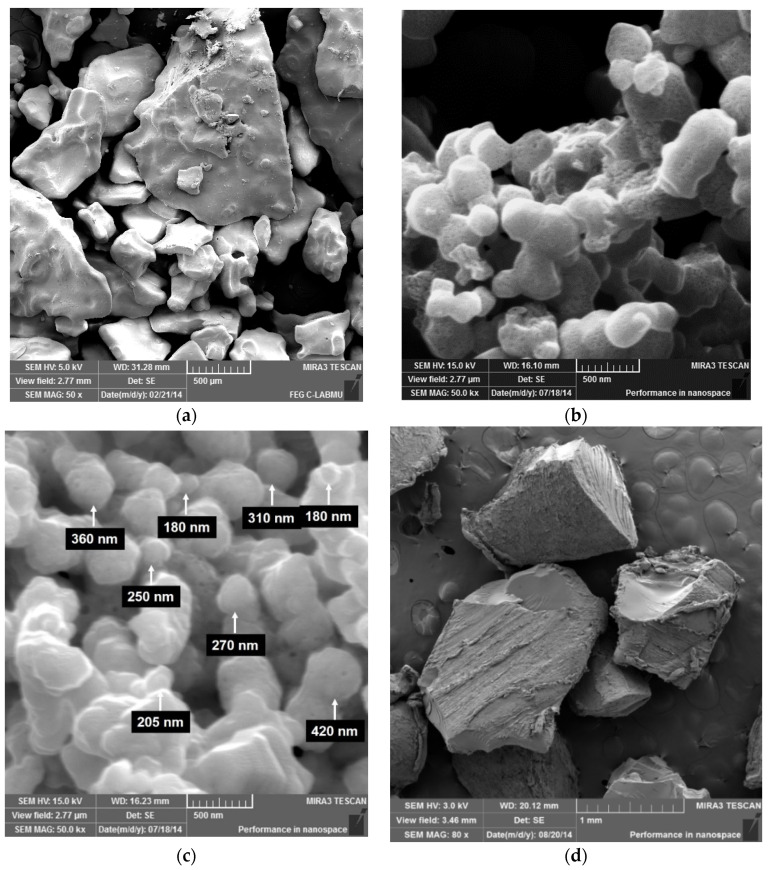
*SEM–FEG* images of as–received AgNO_3_ (**a**); Ag_2_O resulting from the oxidation of AgNO_3_ (**b**); (Ag) resulting from the reduction Ag_2_O (**c**); as–received PVA (**d**).

As–received materials and precipitates resulting from chemical reactions and samples of materials were also characterized by images obtained by *SEM–FEG*. The as–received AgNO_3_, the Ag_2_O, and Ag chemical reaction results and the as–received PVA are illustrated in Figure 3 from a to d, as follows.

Figure 3 shows that the as–received AgNO_3_ sample has irregularly shaped particles and variable size distribution; however, close to the millimeter scale. It can also be observed that after the chemical reactions, both the intermediate product, silver oxide, and the product, Ag, have a morphology that resembles spheres and with a more homogeneous size than the initial material. Similarly, it shows that the chemical reactions caused a severe refinement of the particles since they are submicrometric, and several of them are between 200 and 400 nanometers in size. The as–received PVA proved to have an irregular and approximately equiaxial morphology with an approximate size of 1 mm. 

### 3.4. SEM/FEG of Milled PVA

**Figure 4:**
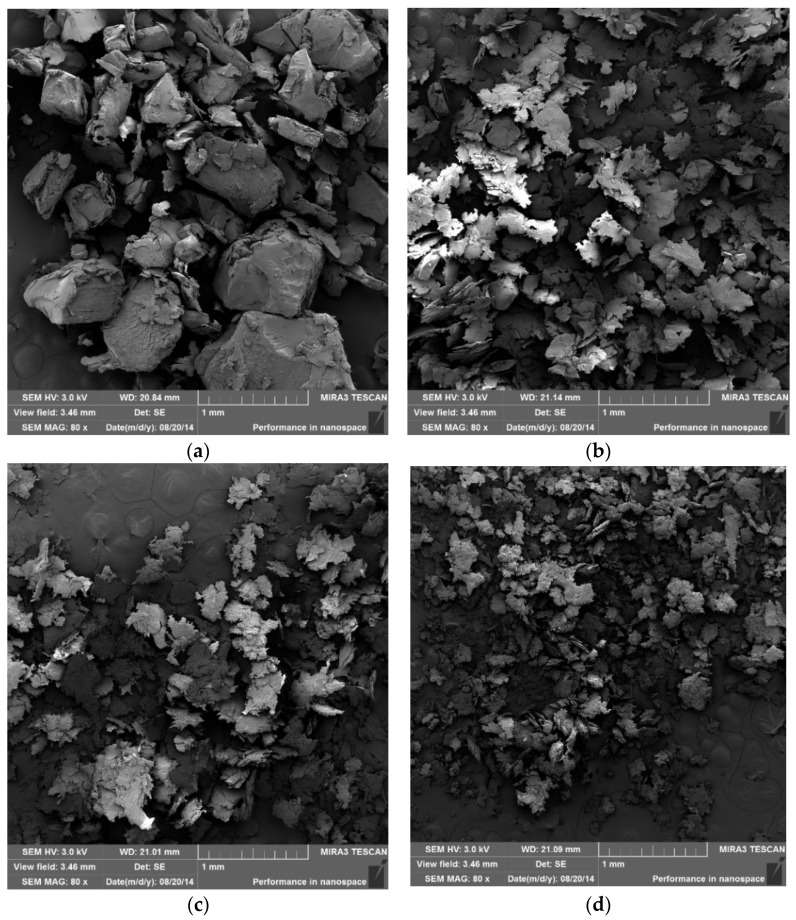

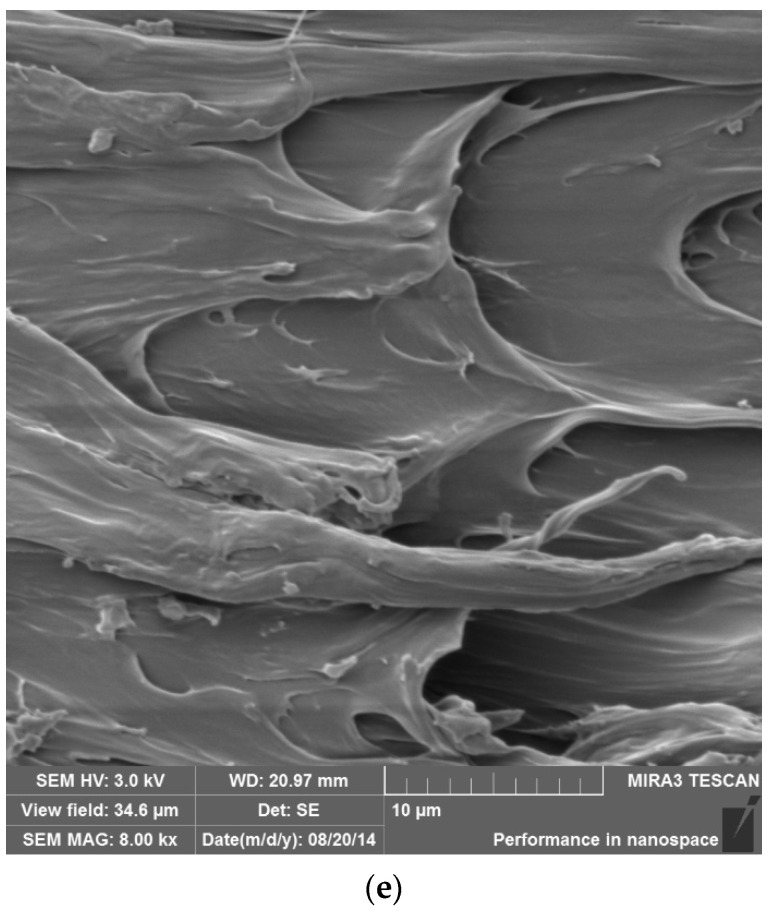
*SEM–FEG* of PVA after milling in an Attritor–type mill: (**a**) PVA milled for 0.5 h; (**b**) PVA milled for 2 h; (**c**) PVA milled for 4 h; (**d**) PVA milled for 16 h; (**e**) surface detailed image from PVA milled for 4 h.

The PVA samples were performed in an Attritor mill with milling parameters described on Table 1 to verify the effects of milling on particle size and shape. Afterwards, the research was conducted using *SEM/FEG*. Finally, the results of the ground PVA samples were compared with the as–received material (Figure 3d). Figure 4 illustrates the size and morphology of the as–received, and milled PVA particles.

Figure 4 compares the effects of mechanical alloying milling time on the PVA size and morphology, for several conditions in an Attritor–type mill.

A comparison of Figure 3d and Figure 4a–c, shows that the PVA begins to decrease in size with respect to the as–received material from the initial stages of milling. However, after 30 min of milling, not all particles underwent a size reduction process. After 1 h of milling, a greater homogeneity in the size of the particles can be observed. There are no significant differences in size and morphology between using a timespan of 2 h and 4 h of milling. The longest the timespan, the smallest the polymer particles achieved. Another observation made through the detailed image (e) is that for 4 h milling, the PVA particles undergo welding and fracture processes, typical of ductile materials [1,2]. In addition, the approximately equiaxial shape of the as–received material results in the form of thin, flat flakes [1,2]. This type of behavior has been previously reported for other kinds of polymers [6].

### 3.5. Characterization by Differential Exploratory Calorimetry (DSC) of the Poly(vinyl Alcohol) Ground at Different Times

**Figure 5:**
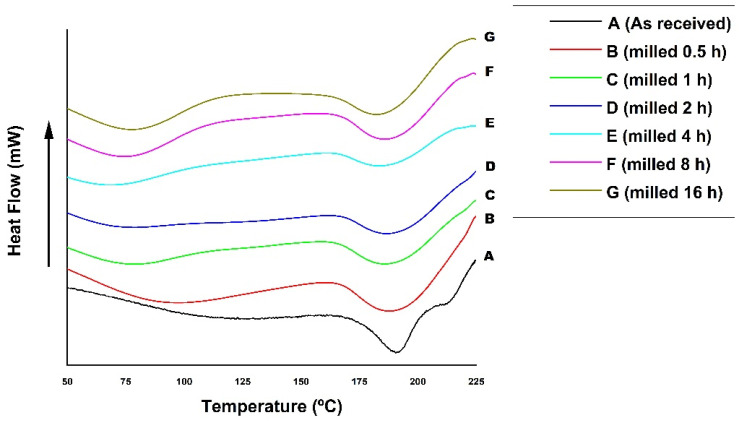
Differential Exploratory Calorimetry Curve (DSC). PVA in several milling conditions compared to the as–received material.

Figure 5 shows the effect of mechanical alloying in PVA characterized by *DSC*.

The polymer’s melting temperature was determined by analyzing the heat flow versus the temperature curve. Table 6 contains these values for each processing situation.

Figure 5 shows the effects of the mechanical alloying on the melting temperature of the PVA with respect to the as–received material. *DSC* can determine first–order transitions that involve enthalpy variation (endothermic and exothermic events), such as melting temperature. The polymer’s melting temperature is measured at the deepest point of the endothermic peak corresponding to temperature [7]. They recall that the physical characteristics of the polymer depend on the degrees of hydrolysis and polymerization [7,28,30,31,56]. It was observed that the milling leads to a decrease in the melting temperature from the as–received sample. However, this decrease is not significant (a difference of approximately 5% between the as–received material and the ground for 16 h). Nevertheless, this comparative effect can still be seen on the peaks around 190 °C in Figure 5, illustrating that the melting temperature of the material ground for 4 h was lower than that of the as–received sample. This effect may be related to the decrease in size and change in the morphology of the PVA particles (a form of thin, flat flakes). Consequently, a greater surface area may favor a faster and more efficient heat exchange, decreasing the polymer’s melting temperature. The PVA melting temperature values listed in Table 6, whether as–received or processed, are within the same range mentioned in the literature [7,28,30,31,56].

Based on the preliminary results described in item 2.6 and characterizations by *SEM–FEG* and *DSC*, the selected condition was processing the PVA for 4 h before the manufacture of films by hot pressing. There is a refinement of the material in this processing time and consequently a decrease in its melting temperature. In addition, it does not take too long to achieve well dispersed Ag particles, which justifies the preferred choice.

### 3.6. Mixing PVA and Silver in an Attritor Mill and Fourier Transformed Infrared (FTIR) Spectra of Powder Mixtures 

**Figure 6:**
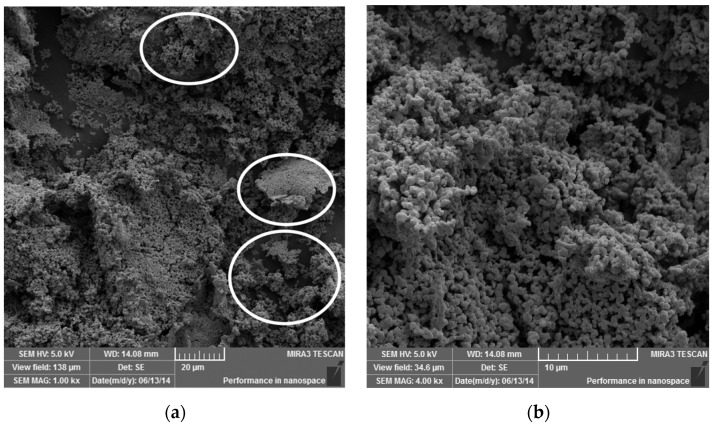
*SEM–FEG* images of composite PVA/Ag obtained by mechanical alloying. In (**a**), some flakes of PVA are highlighted with white circles, which are impregnated with Ag that can be better observed in (**b**) with higher magnification.

Figure 6a,b illustrates the result of the mixture of PVA and 10% wt. Ag by mechanical alloying in an Attritor–type mill for 4 h.

When comparing Figure 4c and Figure 6a, it is still possible to observe the presence of flakes, which are circled with a white line. With a more detailed magnification, it is possible to see that the flakes are impregnated with Ag particles.

**Figure 7:**
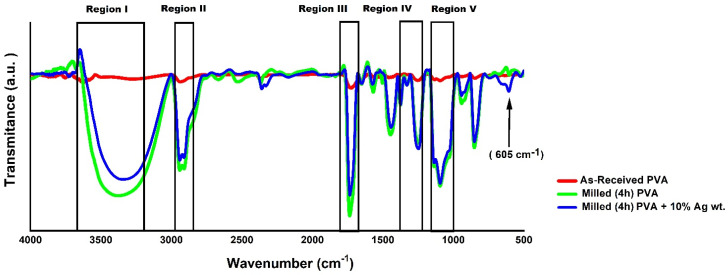
*FTIR* spectra of as–received PVA powder samples, PVA mechanical alloyed for 4 h, with 10% wt. Ag and without Ag.

In Figure 7, the *FTIR* spectra of the as–received PVA and mechanical alloyed PVA powders with 10% wt. Ag and without Ag can be observed on the same scale. 

By analyzing Figure 7, five regions are highlighted in the spectra, corresponding to the interactions of some types of connections at specific *FTIR* frequencies (cm^−1^). These five regions are summarized in Table 7.

Figure 7 shows that the PVA sample milled with 10% wt. Ag presents a band near 605 cm^−1^, indicated in the graph with an arrow, different from the as–received material. This band may suggest a chemical interaction between PVA and Ag particles due to the far–infrared region, meaning stretching and bending vibrations of bonds between metal atoms and both inorganic and organic ligands at frequencies lower than 650 cm^−1^ [57,58,59].

**Figure 8:**
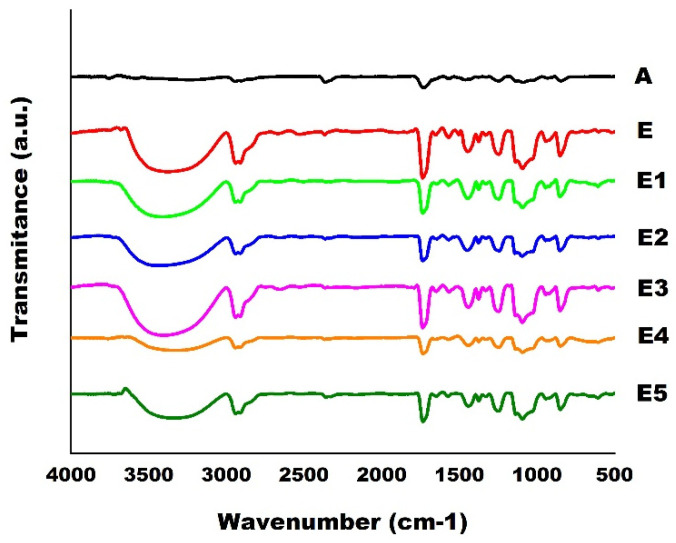
*FTIR* spectra of as–received PVA and milled powder samples by mechanical alloying with and without Ag in several studied conditions. (A) as–received PVA; (E) Pure PVA milled for 4 h; (E1) PVA + 0.5% wt. Ag milled for 4 h; (E2) PVA + 1% wt. Ag milled for 4 h; (E3) PVA + 2.5% wt. Ag milled for 4 h; (E4) PVA + 5% wt. Ag milled for 4 h; and (E5) PVA + 10% wt. Ag milled for 4 h.

In Figure 8, each detailed spectrum can be observed separately, in an appropriate scale, for some of the conditions studied (Table 2 and Table 3).

The analysis of the as–received PVA spectrum in Figure 8, showed a narrow band with a maximum peak at 3626 cm^−1^ representing free O–H bonds from the alcohol functional group. For samples ground from pure PVA and PVA with Ag particles, changes in this bandwidth and amplitude (% transmittance) concerning as–received PVA may be related to the fact that the milling has promoted stretching of the connections between O–H. However, Ag particles did not influence these functional group interactions [7,30,31,56,59,60,61,62,63].

The evidence is the lack of significant differences between milled samples with and without Ag (see Figure 7 in Region I). The maximum peaks were approximately at the frequency of 3300 cm^−1^. Region II, shown in Figure 7, comprises two situations involving stretching of the C–H bonding. The first situation is related to the symmetrical stretch with a maximum peak at 2937 cm^−1^. The second situation is associated with the asymmetry of the region with a maximum height of 2910 cm^−1^. It was noted that this effect occurred in the milled material with and without Ag particles while it was not evidenced in the as–received material. The values of these peaks for all situations studied can be seen in detail in Figure 8. 

Region III, shown in Figure 7, with a band between 1735 and 1750 cm^−1^, which can be seen in greater detail in Figure 8, corresponds to the C=O bonding. The maximum peak is around 1740 cm^−1^. The origin of this functional group is polyvinyl acetate, which is the raw material for obtaining PVA Region IV, highlighted in Figure 7 and seen in detail for each situation in Figure 8, corresponds to a frequency range from 1200 to 1465 cm^−1^. Two peaks appear with maximum values of 1237 and 1438 cm^−1^ as they conform to the C–O–H bond, coupled with the CH_2_ vibrations of the methylene group. 

In Region V, Figure 7 corresponds to the stretching vibration of the C–O bonds of the carbonyl group. Two overlapping peaks appear with maximum heights at 1090 and 1200 cm^−1^. The details of this region can be observed for all of the situations studied in Figure 8. Transmittance was verified to be 99% on as–received PVA, while in the conditions with milled PVA, the transmittance ranged from 75 to 85%. Therefore, there was no evidence of the influence of Ag levels in this situation, in general, confirmed through the analysis of Figure 8, the difference in peak intensity between the as–received PVA and the PVA milled by mechanical alloying. This effect may be related to the increased surface area or the polymer’s degree of degradation caused by the milling process. However, the observed bands are in the same range of values described in the literature. In addition, no significant difference was found when comparing ground PVA alone with ground PVA with various levels of Ag particles, using the same processing conditions. [7,30,31,56,59,60,61,62,63].

### 3.7. Films Preparation by Hot Pressing and Characterization by Optical Microscopy 

**Figure 9:**
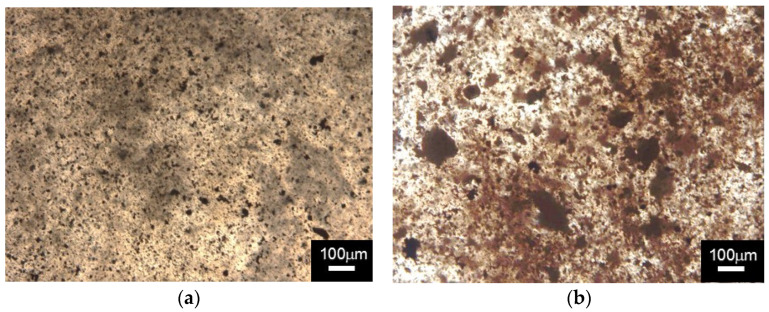
Optical Microscopy of the films produced from mechanically alloyed powders for 4 h. This is (**a**) PVA + 2.5% wt. Ag and (**b**) PVA + 10% wt. Ag.

The samples used to prepare films were: as–received PVA (A), pure PVA milled for 4 h (E) from Table 2, and powdered mixture PVA with Ag particles, from Table 3 (E1 to E5). Hot pressing films with 0.3 mm thickness and around 70 mm diameter were made from the powders obtained with 4 h of mechanical alloying in an Attritor mill. Although the information is not objective, nor appealing, the surface quality of the film formed by the previously milled material proved to be abundantly higher than that of the as–received material, comparing the exact parameters of time, temperature, and pressure in the pressing process. Figure 9 illustrates PVA films with 2.5% wt. Ag (a) and 10% wt. Ag (b).

Figure 9, shows, by means of light microscopy, a number of dark spots on the surface of the films that contained Ag. As a result, the distribution of Ag particles on the PVA matrix surface could be understood. The particles are fine and evenly distributed over the entire length of the film for mass fractions up to 2.5% wt. Ag. For the 10% wt. Ag, although the Ag is still well distributed in the film, some larger particles are noted, suggesting agglomeration.

### 3.8. Characterization of the Fracture Surface of the Films by SEM–FEG

**Figure 10:**
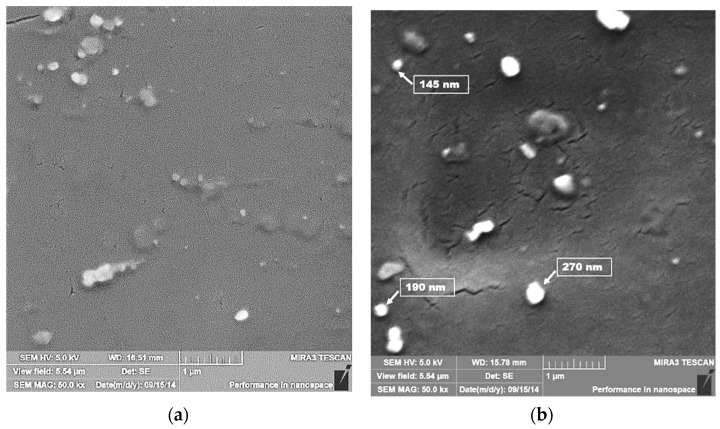
*SEM–FEG* of the film’s fracture surface produced by mechanical alloying powder for 4 h. (**a**) PVA + 2.5% wt. Ag and (**b**) PVA + 10% wt. Ag.

The fragility of the PVA films was conducted in a liquid nitrogen bath, for 5 min, and caused them to break with the aid of pliers. The objective was to verify the distribution of Ag particles and their thickness. As the surface to be observed was rather thin, the analysis was positioned at the edge of the support, fixed with carbon tape, and later metalized with a gold and palladium alloy. The results for the addition of 2.5% wt. Ag and 10% wt. Ag are shown in Figure 10.

In Figure 10, the fracture surface observed (in the thickness of the film) was analyzed by *SEM–FEG.* The occurrence of shiny and spherical particles, like the Ag images analyzed in Figure 2 is worth noting. Some of these particles are micrometric, probably due to the aggregation of Ag during processing. However, relatively ultrafine particles [64,65] are observed, with a diameter of 200 nanometers. Another observation is that these particles are distributed on the film thickness for all Ag proportions. It is significant to note that the contrast of the images is shallow because the energy of the electron beam is also low (5 kV according to the scale bar). It is appropriate to recall that greater energies would degrade the polymeric material.

### 3.9. Chemical Microanalysis of the Particle in the Polymeric Film by Energy Dispersive Scanning (EDS)

**Figure 11:**
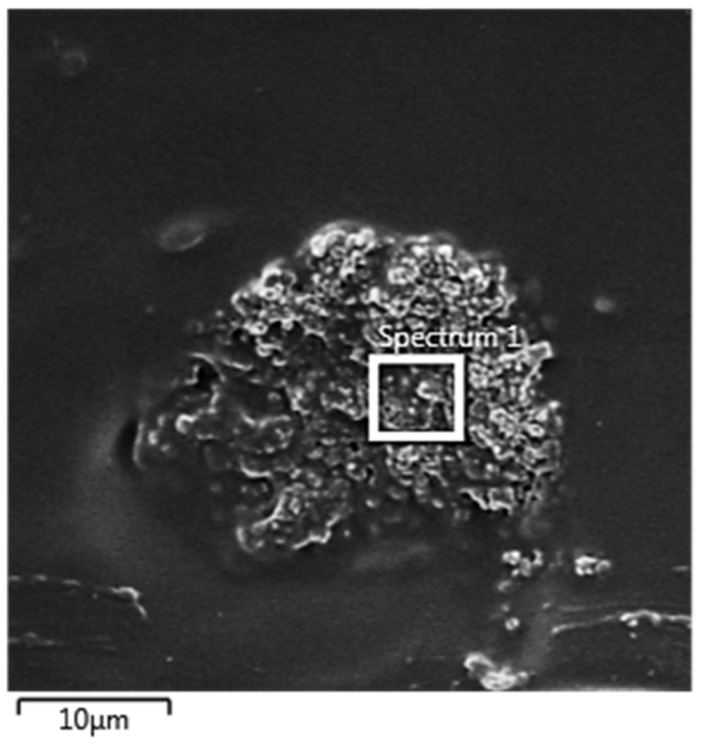
*SEM–FEG* of agglomerated particles in the PVA film with 10% wt. Ag.

The *EDS* detected that the ultrafine particles were Ag, thus averting contamination in the film. The results for all samples were similar. Figure 11 shows an image of agglomerated particles observed in the PVA film with 10% wt. Ag. Table 8 shows its chemical microanalysis.

Figure 11 shows the agglomerated particles observed in the PVA film with 10% wt. Ag. A revision of the chemical microanalysis listed in Table 8, evidences the presence of Ag. The presence of gold and palladium is due to the metallization of the film surface.

### 3.10. Tests of Microbiological Activity of Polymeric Films

**Figure 12:**
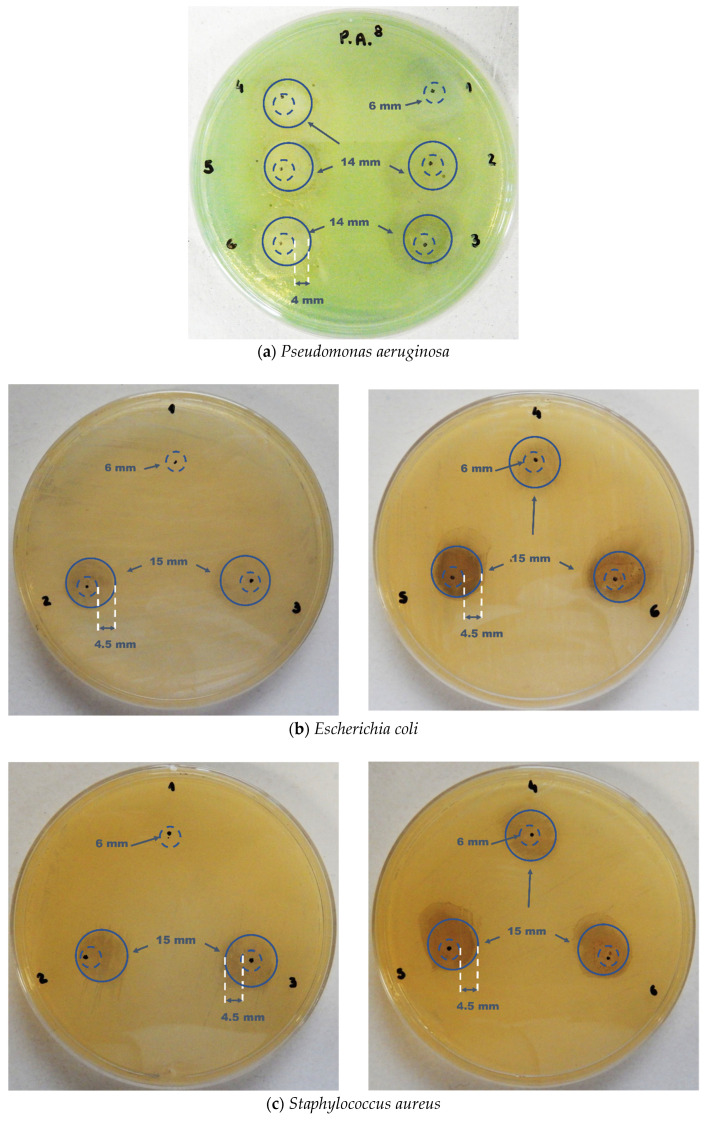

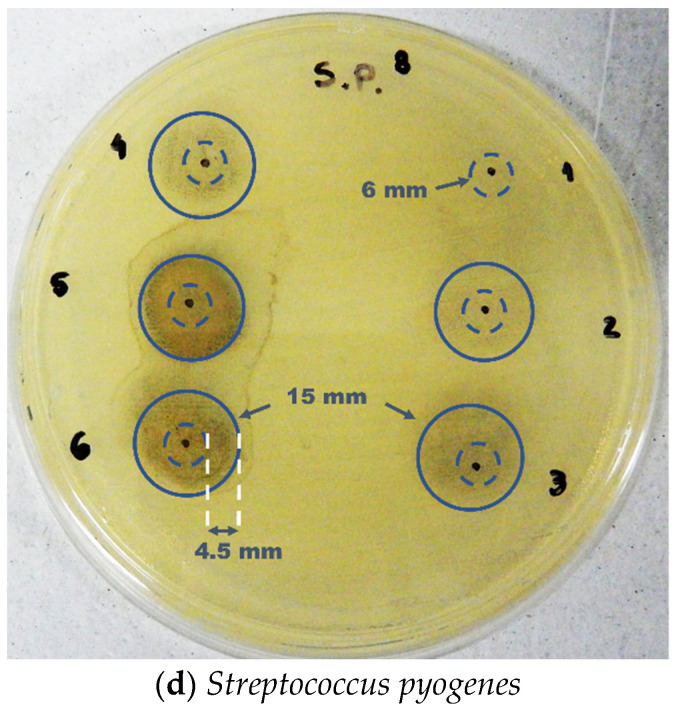
Photographs of the plates sown after incubation for 24 h in an oven at 36 °C. (**a**) *Pseudomonas aeruginosa*; (**b**) *Escherichia coli*; (**c**) *Staphylococcus aureus* and (**d**) *Streptococcus pyogenes*. The numbers correspond to the films under the following conditions: (1) pure PVA; (2) PVA + 0.5% wt. Ag; (3) PVA + 1% wt. Ag; (4) PVA + 2.5% wt. Ag; (5) PVA + 5% wt. Ag; and (6) PVA + 10% wt. Ag. Outer circles represent the inhibition zone, the inner dotted circles represent the polymeric film, and the distance between the inner and outer circles represents the inhibition halo.

The antimicrobial activity of the films produced was determined by the agar diffusion method, a fast and straightforward technique indicated to verify the activities of the film samples in their different concentrations [32,35,37,41,42,44,45,46,47,48,49,50,55]. Figure 12 illustrates the culture plates on Müller Hinton agar seeded with the bacteria in the following sequence: (a) *Pseudomonas aeruginosa*; (b) *Escherichia coli*; (c) *Staphylococcus aureus*; and (d) *Streptococcus pyogenes*. The numbers indicated on the plates correspond to pure PVA and PVA films with different concentrations of Ag particles, as described in Table 4.

Figure 12 illustrates the absence of antimicrobial activity observed only in the pure PVA film (condition 1 for all plates), which has also been reported in the literature [49]. There was no bacterial growth on the films in the other conditions (2 to 6). Likewise, no colonies developed either. In addition, from 1% wt. Ag, particles concentration (conditions 3 to 6), an irregularly shaped inhibition halo was formed, like that observed in the literature [60]. The film’s affinity can explain this irregularity with the residual moisture of the culture medium. The polymeric film obtained was highly hydrophilic, which may be due to the dry production process without using additives that decrease the material’s solubility in water [60]. 

As can be seen from Figure 12a, the PVA film with Ag particles showed inhibition of the growth of strains of *P. aeruginosa*, one of the most antibiotic–resistant bacteria. An inhibition zone near to 15 mm and an inhibition halo of approximately 4 mm occurred for conditions 3 to 6. Comparing the expected inhibition zone for the composite of PVA and Ag nanoparticles [49], which would measure between 9 and 11 mm, the results are close to those found herein. In this study, the ultrafine Ag particles did not reach the size range to be considered nanometric [64,65], but even so, they showed good antimicrobial activity compared to other studies [41,50,55]. By analyzing Figure 12b–d, there was no bacterial growth on any of the tested films, except in the control one, consisting only of PVA, without incorporating the particles. This information is of paramount importance as it indicates that the material developed may protect against bacterial growth. Thus, it would potentially imply local protection against invasion by microorganisms, a fact demonstrated in the tests performed, making the process a promising path for the production of surface protective polymeric films.

## 4. Conclusions

Based on the results discussions, it can be concluded that:

X-ray diffraction measurements showed that it was possible to obtain Ag_2_O from the oxidation of AgNO_3_ with 1 molar NaOH solution and subsequently obtain Ag by reducing Ag_2_O with formaldehyde. In addition, *SEM–FEG* measurements showed that many of these Ag_2_O and Ag particles were ultrafine, measuring between 200 and 400 nanometers.

It was observed that the mechanical alloying promoted significant refinement and a morphology change in the PVA particles, changing in shape close to the equiaxial of the as–received material to fine flakes after milling. The milling proved to be a promising path to follow for distributing Ag in the PVA matrix in a homogeneous manner throughout the thickness of the polymeric film.

The agar antimicrobial susceptibility test evaluated the films produced. It was observed that there was no inhibition of microbial activity in pure PVA. The inhibition was verified on the surface of all the films that had Ag particles and for all the bacteria tested.

## Figures and Tables

**Table 1:** Milling Parameters.

Ball Size (mm)	Milling Power	Speed Rotation (rpm)	Milling Temperature
6.35	38:1	600	Water cooled at room temperature

**Table 2:** Milling time condition of commercial PVA from the as–received material.

Sample	Condition
A	As–received
B	Milled for 0.5 h
C	Milled for 1 h
D	Milled for 2 h
E	Milled for 4 h
F	Milled for 8 h
G	Milled for 16 h

**Table 3:** Weight % fractions of (Ag) and Poly(vinyl alcohol) (PVA).

Samples	Condition
A	As–received PVA
E1	PVA + 0.5% wt. Ag
E2	PVA + 1% wt. Ag
E3	PVA + 2.5% wt. Ag
E4	PVA + 5% wt. Ag
E5	PVA + 10% wt. Ag

**Table 4:** Samples used to assess the antimicrobial activity of polymeric films.

Samples	Condition Films
1	As–received PVA
2	PVA + 0.5% wt. Ag
3	PVA + 1% wt. Ag
4	PVA + 2.5% wt. Ag
5	PVA + 5% wt. Ag
6	PVA + 10% wt. Ag

**Table 5:** Values of the lattice parameters of silver nitrate (AgNO_3_), silver oxide (Ag_2_O), and silver (Ag).

Samples	Lattices (nm)		
	a	b	c
AgNO_3_	0.699	0.733	1.013
Ag_2_O	0.471	0.471	0.471
Ag	0.409	0.409	0.409

**Table 6:** Melting temperature values obtained by Differential Scanning Calorimetry (*DSC*) for each sample of PVA.

Processing Condition	Melting Point (°C) Measured by *DSC*
As–received	191
Milled for 0.5 h	188
Milled for 1 h	187
Milled for 2 h	187
Milled for 4 h	185
Milled for 8 h	185
Milled for 16 h	182

**Table 7:** A list of the five regions highlighted in the spectrum illustrated in Figure 7 corresponds to some types of bonding interactions at specific *IV–TF* frequencies (cm^−1^).

Region	Type of Bonding	Frequency Ranges (cm^−1^)
I	O–H	3200–3650
II	C–H	2800–2900
III	C = O	1700–1750
IV	C–O–H e CH_2_	1230–1465
V	C–O	1000–1200

**Table 8:** Chemical elements detected by *EDS*.

Chemical Elements	
Elements	% wt.
Ag	92.47
O	4.81
Au	1.8
Pd	0.92

## Data Availability

Not applicable.
